# Cell line-based *in vitro* models of normal and chronic bronchitis-like airway mucosa to study the toxic potential of aerosolized palladium nanoparticles

**DOI:** 10.3389/fmed.2024.1422792

**Published:** 2024-10-08

**Authors:** Jie Ji, Katja Jansen, Vadim Kessler, Gulaim Seisenbaeva, Per Gerde, Maria Malmlöf, Lena Palmberg, Swapna Upadhyay

**Affiliations:** ^1^Unit of Integrative Toxicology, Institute of Environmental Medicine, Karolinska Institutet, Stockholm, Sweden; ^2^Inorganic Bionanotechnology Unit, Department of Chemistry and Biotechnology, Swedish University of Agricultural Sciences (SLU), Uppsala, Sweden; ^3^Inhalation Sciences Sweden AB, Stockholm, Sweden

**Keywords:** animal alternative models, nanoparticle exposure, 16HBE/cell line, inflammation, oxidative stress

## Abstract

**Background:**

Physiologically relevant cell line-based models of human airway mucosa are needed to assess nanoparticle-mediated pulmonary toxicity for any xenbiotics expsoure study. Palladium nanoparticles (Pd-NP) originating from catalytic converters in vehicles pose health risks. We aimed to develop *in vitro* airway models to assess the toxic potential of Pd-NP in normal (Non-CB) and chronic bronchitis-like (CB-like) mucosa models.

**Methods:**

Bronchial mucosa models were developed using Epithelial cells (16HBE: apical side) co-cultured with fibroblast (basal side) at an air-liquid interface. Furthermore, both Non-CB and CB-like (IL-13 treatment) models with increased numbers of goblet cells were used. The models were exposed to 3 different doses of aerosolized Pd-NP (0.2, 0.3, and 6 μg/cm^2^) using XposeALI^®^ and clean air as a control. After 24 h of incubation, the expression of inflammatory (*IL6*, *CXCL8*, *TNFα*, and *NFKB*), oxidative stress (*HMOX1*, *SOD3*, *GPx*, and *GSTA1*), and tissue injury/repair (*MMP9/TIMP1*) markers was assessed using qRT–PCR. The secretion of CXCL-8 and the expression of a tissue injury/repair marker (MMP-9) were measured via ELISA.

**Results:**

Significantly (*p* < 0.05) increased expressions of *CXCL8*, *IL6*, and *NFKB* were observed at the highest dose of Pd-NP in CB-like models. However, in Non-CB mucosa models, a maximum effect on *TNFα* and *NFKB* expression was observed at a medium Pd-NP dose. In Non-CB mucosa models, *SOD3* showed a clear dose-dependent response to Pd-NP exposure, while *GSTA1* expression was significantly increased (*p* < 0.05) only at the lowest dose of Pd-NP. The secretion of CXCL-8 increased in a dose-dependent manner in the Non-CB mucosa models following exposure to Pd-NP. In CB-like models, exposure to high concentrations of Pd-NP significantly increased the release of MMP-9 compared to that in other exposure groups.

**Conclusion:**

The combination of our Non-CB and CB-like mucosa models with the XposeALI^®^ system for aerosolized nanoparticle exposure closely mimics *in vivo* lung environments and cell-particle interactions. Results from these models, utilizing accessible cell lines, will maximize the reliability of *in vitro* findings in human health risk assessment.

## Introduction

The lungs, interfaced with the external environment for gas exchange, are constantly exposed to inhaled agents such as cigarette smoke, occupational dusts, and air pollutions. These exposures have far-reaching consequences, from reduced lung function and respiratory sensitization in childhood to increased morbidity and mortality in adulthood, encompassing cancer, cardiovascular disorders, and airway diseases such as COPD (1).

COPD is a chronic condition affecting up to 10.6% of the global population with approximately 3 million deaths annually (2), often manifests as chronic bronchitis, which is primarily linked to smoking. Notably, short-term exposure to air pollutions and long-term exposure to atmospheric particulate matter (PM) have also been implicated in chronic bronchitis (3, 4). Moreover, the increasing exposure of engineered nanoparticles, propelled by their exponentially increasing utilization owing to their unique properties compared to those of bulk materials (e.g., magnetism, thermal conductivity, and catalytic capabilities), is another emerging concern.

Metallic-based nanomaterials have attracted great scientific attention in the nanotechnology field. For instance, palladium (Pd), a rare and precious metal that belongs to the platinum group of elements (5), is largely employed as an active catalyst material in automotive catalytic converters. Moreover, Pd-NP are frequently used in nano-medicine and nano-diagnostics due to their efficient and broad catalytic activities, particularly in chemical reactions of therapeutic relevance (6). Increasing amounts of airborne platinum group elements (PGE) emitted by vehicle exhaust catalysts, including Pd-NP, have been measured in ambient air (7–9). One study demonstrated that the main route of PGE uptake in the human body is via respiratory exposure rather than ingestion (10). According to the World Health Organization’s Environmental Health Criteria document on Palladium (EHC 226, 2002), the daily Pd uptake via inhalation is approximately 2.2 ng per person (11). Although previously assumed to pose minimal health risks, emerging evidence suggests that environmental exposure to PGE may present significant health concerns, particularly at chronic, subclinical levels (12). To better assess the risk of airborne substances such as PGE to human health, both large-scale epidemiological monitoring and sophisticated research models are needed.

According to the Organization for Economic Co-operation and Development (OECD), acute inhalation toxicity studies, which aim to define both local and systemic toxicity, are currently conducted using OECD Test Guidelines (TG 403 (Acute Inhalation Toxicity), TG 436 (Acute Toxic Class Method), and TG 433 (Fixed Concentration Procedure)). These guidelines are all based on exposing rodents to test compounds (gas, vapor, aerosol, or mixture) (13). However, these approaches are limited by interspecies differences in respiratory tract architecture and function relative to humans. Moreover, the relative size of inhaled particles differs significantly between rodents and humans, hindering conclusions on human hazard potential (13). The use of animal models is further restricted by attempts to alleviate animal suffering and to save costs.

Human cell cultures provide an alternative tool that conforms to the principles of the 3Rs (i.e., replacement, reduction, and refinement of animal use) and circumvents the uncertainty of interspecies extrapolation (14). In traditional *in vitro* models, however, cells are typically fully submerged in a culture medium supplemented with the agent of interest. *In vivo,* exposure of the lungs, on the other hand, occurs at the air-liquid interface (ALI). The apical surface of the ciliated columnar epithelium is only covered by a thin endogenously produced two-layer liquid. The outer mucus layer provides the first physical barrier that traps inhaled particles that are continually transported toward the pharynx via the underlying layer (15). The lower layer of the peri-ciliary fluid has a low viscosity, which allows proper functioning of this mucociliary escalator (16). Submerged cultured cells develop a squamous epithelial phenotype without correct basolateral to apical orientation, ciliation, or mucus production, which is not representative of the *in vivo* situation.

For this reason, recent approaches have progressively integrated ALI systems to study particle-cell interactions under more physiological conditions (17). For instance, commercially available cell exposure systems, such as ALICE, Vitrocell, and Cultex^®^ RFS, have been used (18–20). Additionally, we previously developed a sophisticated XposeALI^®^ 3D cell exposure module (21). The XposeALI^®^ combines aerosol capability with 3D cell models cultured at an air-liquid interface, which enables studies of the cellular effects induced by airborne particles. Furthermore, the construction of a 3D cell model and co-culture systems enables cell-matrix and cell–cell interactions, which are crucial for a greater resemblance to the physiological setting. Lung tissue consists of more than 40 different cell types, and to mimic a reliable *in vivo* microenvironment, *in vitro* models engineered for toxicological or pharmacological applications must include specialized cells that are assembled in a realistic architecture (22, 23). Pulmonary fibroblasts maintain structural integrity and tissue homeostasis by producing extracellular matrix (ECM) and growth factors. Moreover, they actively participate in inflammatory responses via the local release of cytokines (24). Co-cultures with tissue-specific cells have been shown to result in cellular cross-talk and more sensitive responses (25).

In previous studies, we developed advanced physiologically relevant *in vitro* models with primary bronchial epithelial cells, which have great *in vivo* resemblance (21, 26). However, access to primary cells is often limited, whereas lung cell lines (e.g., 16HBE14o, a human bronchial epithelial cell line) have the potential for large-scale toxicity screenings due to their easy availability and ability to expand. Therefore, in this study, we developed and evaluated normal (Non-CB) and chronic bronchitis-like (CB-like) mucosa models using the 16HBE cell line and measured inflammatory and oxidative stress markers at both the mRNA and protein levels after exposure to airborne Pd-NP. Using the same Pd-NP as in our primary bronchial mucosa models at ALI (21) and submerged (27), we were able to compare the exposure response in these different models.

## Materials and methods

### Model establishment

#### Culturing of the human bronchial epithelial cell line (16HBE cells)

16HBE cells (SCC150, Sigma–Aldrich, MD, United States) were originally derived from the second-generation bronchi of a 1-year-old male heart-lung transplant patient and transformed into an epithelial cell line that shows basal cell characteristics. Cell culture plasticware (Petri dish, Thermo Fisher Scientific, MD, United States) was precoated for 2 h with 30 μg/mL Collagen vitrogen 100 (Cohesion Technologies, Palo Alto, CA, United States), 10 μg/mL fibronectin (Gibco, Paisley, Scotland, United Kingdom), 10 μg/mL bovine serum albumin (BSA; Boeringer Mannheim, Mannheim, Germany) and 20 U/mL penicillin/streptomycin (PEST; BioWhittaker, Walkersville, MD, United States) in phosphate-buffered saline buffer without calcium and magnesium (PBS; Gibco, Thermo Fisher Scientific, MD, United States). The cells were cultured on precoated plasticware using Dulbecco’s modified Eagle’s medium (DMEM; Gibco, Thermo Fisher Scientific, MD, United States) supplemented with 10% fetal bovine serum (FBS; Gibco, Thermo Fisher Scientific, MD, United States) and 20 U/mL PEST under submerged conditions. 16HBE cells were cultured to 80% confluency at 37°C in a humidified atmosphere of 5% CO_2_. The medium was changed every second day. 16HBE cells were transferred from plasticware to transwell inserts in 12-well plates to establish cell line-based bronchial mucosa models as described previously using human primary bronchial epithelial cells (21, 28).

#### Culture of human lung fibroblasts (MRC-5)

MRC-5 (Medical Research Council cell strain 5) cells are lung fibroblasts originally derived from a 14-week-old male fetus. MRC-5 cells obtained from American Type Cell Culture (passage 27) were submerged in a Petri dish with DMEM supplemented with 10% FBS, 1% nonessential amino acids, 1% HEPES, and 20 U/mL pen/strep (21, 28). MRC-5 cells were added to the basal side of the insert to establish a co-culture bronchial mucosa model as described below.

#### Establishment of a co-cultured bronchial mucosa model

**Figure 1:**
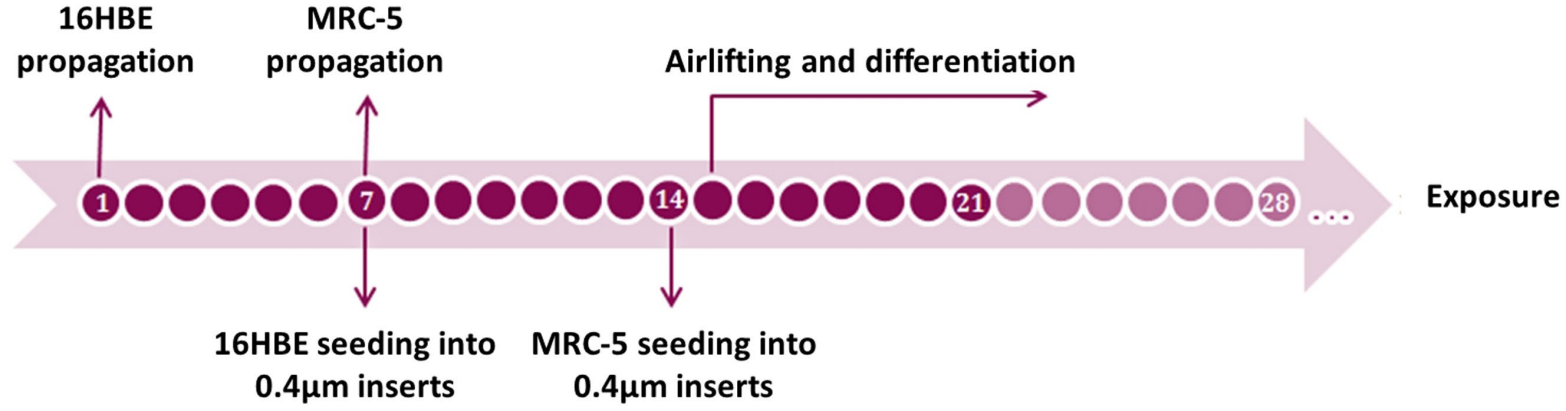
Timeline of the 16HBE ALI model set up. Differentiation should take at least 7 days and can be kept on until 2 weeks after airlifting.

Generation of the co-cultured airway mucosa models took 21–36 days, including cell propagation, cell culture on transwell inserts, airlifting, and cell differentiation, as previously described (21, 28) using primary bronchial epithelial cells (PBECs) (Figure 1). First, 16HBE cells were propagated under submerged conditions in pre-coated cell culture plasticwares, as described above. The cells were trypsinized when they reached more than 80% confluency. Finally, the cells were seeded on pre-coated, semi-porous 0.4 μm transwell inserts in 12-well plates at a seeding density of 10^5^ cells/insert. After reaching confluency around day 7, the inserts were turned upside down and placed in a sterile petri dish, and MRC-5 cells were added to complete DMEM on the basal side of the insert membrane. The petri dish was covered and incubated for 30 min at 37°C; 50 μL of complete DMEM was added every 10 min to prevent draining. After a total of 1 h of incubation, the inserts were again placed in the plate with the addition of 1 mL of complete DMEM to each insert, both at the apical and basal sides of the inserts, to cover the cells on both sides. After overnight incubation, the models were airlifted by removing the medium and adding 875 μL co-culture medium (i.e., complete DMEM supplied with 6 μg/mL CaCl_2_ in ddH_2_O, 15 ng/mL ethanolamine in ddH_2_O, and 10^−5^ M retinoic acid) to the basal side of the insert. After airlifting, the models were maintained for 1–3 weeks. To establish a model of chronic bronchitis (CB-like) mucosa, 1 ng/mL recombinant human IL-13 was freshly added to the co-culture medium only on the basal side of the insert and maintained for 2 weeks during the ALI process. The chosen IL-13 concentration and treatment duration used in present study were adopted from the protocol in our previous paper (21). The airway mucosa models were maintained at 37°C and 5% CO_2,_ and the medium on the basal side was changed every second day. According to our preliminary experiments, the differentiation of 16HBE cells to ciliated and mucous-producing cells took approximately 2 weeks and is regarded as the optimal time point for experiments.

### Model characterization

#### Measurement of transepithelial electrical resistance (TEER)

To determine the integrity of the bronchial mucosa models, TEER were measured with an EVOM Volt Ohm Meter equipped with STX01 electrodes connected to a Millicell ERS voltohm meter (Millipore, Carrigtwohill, Ireland). TEER values were measured at 24 h, 4 days, 1 week, and 2 weeks under ALI conditions with 2 inserts per time point. TEER values were corrected for the background value contributed by the growth medium using the TEER of polyester transwell filter membranes without cells filled with PBS, which was considered a blank insert (without cells). For cells cultured under ALI conditions, the fluid volume was adjusted to 500 μL apically and 1 mL in the basal chamber with pre-warmed medium. TEER was calculated as follows: (TEER value of inserts with cells- TEER value of inserts without cells) x surface area of transwell membrane (0.9 cm^2^) (21, 28, 29).

#### Cell fixation and processing

After finalizing the culturing, the inserts were washed with PBS, and the cells were fixed with 4% formaldehyde overnight at 4°C. The membranes were excised and then processed as follows: dehydration in two changes of 45 min in 95% ethanol and 45 min and 1 h in 99% ethanol, clearing in two changes of 75 min in xylene, and finally embedding in paraffin wax for 4 h and 45 min. After solidification, we cut 5 μm sections using a Microm HM 360 rotary microtome. The sections were transferred onto glass slides and dried at 37°C overnight before staining (21, 29).

#### Histological analysis

The membrane sections were deparaffinized in two steps of 5 min in Ultraclean buffer and rehydrated in two steps of 5 min in 99%, 3 min in 95%, and 3 min in 70% ethanol. After a 3-min washing step with distilled water, sections were stained with Mayer’s hematoxylin solution (HTX; Histolab, Gothenburg, Sweden) for 4 min to visualize nuclei. Subsequently, the sections underwent a 10-min washing procedure using running distilled water to remove excess stain. Then the cytoplasm and other tissue elements we counterstained with a 2% aqueous eosin Y solution (in-house; Sigma–Aldrich, MD, United States) for 2 min, followed by a two-step dehydration of 1 min in 95% ethanol, three steps of 1 min in 99% ethanol and two steps in UltraClear buffer for 1 and 3 min, respectively. Finally, the sections were mounted with Pertex^®^ (Histolab) and photographed under a light microscope.

#### Transmission electron microscopy

For transmission electron microscopic (TEM) characterization, inserts were fixed in 2% glutaraldehyde in 0.1 M sodium cacodylate buffer with 0.1 M sucrose and 3 mM CaCl2 (pH = 7.4). TEM characterizations of both cell models and Pd-NP particles were performed as previously described (21, 29).

#### Immunofluorescence analysis

To visualize ciliation and mucus production, we stained our models with mouse anti-acetylated *α*-tubulin (TUBLIN; Life Technologies, California, US) and a rabbit polyclonal antibody against Mucin 5 AC (MUC5AC; Abcam, Cambridge, United Kingdom). After fixation with 2% formaldehyde and three washes with PBS, the membranes were blocked with PBS containing 1% goat serum, 0.1% Triton and 0.025% sodium azide for 12 min. After washing, TUBLIN (1:200) and MUC5AC (1:100) were added to the blocking buffer, and the membranes were incubated overnight at 4°C. We incubated the membranes with Alexa Fluor 488-conjugated goat anti-mouse (1:800) and Alexa Fluor 555-conjugated goat anti-rabbit (1:400) secondary antibodies for 2 h before they were cut and mounted with Fluoroshield mounting medium supplemented with DAPI (Abcam) for confocal microscopy (21).

### Exposure setup

#### Exposure of Non-CB and CB-like models to Pd-NP using the XposeALI^®^ exposure system

To mimic the *in vivo* exposure of the lung, both the Non-CB and CB-like models were exposed to clean air (sham) or aerosolized Pd-NP (exposed) using the Xpose*ALI*^®^ exposure system as previously described (21, 26). In brief, during each exposure, 3 inserts were placed inside the exposure module. Based on our previous study (21), we observed that the exposure lasting approximately 3 min can effectively clear all particles, preserve the cell model’s viability and responsiveness to exposure. Therefore, we selected 3 min as the high exposure duration and 1 min and 2 min as low and medium exposure durations for titration, respectively. Then exposure to aerosolized Pd-NP was performed for these 3 selected different durations (1, 2, and 3 min) to achieve low, medium, and high Pd-NP concentrations, respectively. The inside of the aerosol holding chamber was covered with wet filter papers, which maintained humidity to maintain cell viability. Additionally, in the exposure module, the inserts, including bronchial models, were always in contact with the basal medium during the exposures to avoid any physiological stress to the cells. Compressed air (100 bars) was used to aerosolize the Pd-NP into the 300 mL holding chamber. The Pd aerosol was removed from the holding chamber at a constant flow rate (120 mL/min) and diverted into triplicate branch exposures at a flow rate of 10 mL/min per branch (21, 26). Following exposure, the cells were removed from the exposure modules, placed in 12-well plates with fresh basal medium, and incubated for 24 h in 5% CO_2_ at 37°C. Both basal medium and cells were collected for further analysis.

To quantify the amount of Pd-NP deposited on the cell surface, three additional inserts without cells were subjected to Pd-NP exposure using the Xpose*ALI*® exposure system for identical durations (1, 2, and 3 min). Subsequently, the membrane of each insert was dissolved in aqua regia, supplemented with a concentrated (25%) ammonia solution. Inductively coupled plasma-mass spectrometry (ICP-MS) was performed to measure the Pd-NP deposition levels on the empty insert with isotopically enriched ^108^Pd as a standard (30). Then by detecting the levels of Pd-NP on the empty inserts, we could determine the total amount of Pd-NP deposited on the cell surface indirectly (μg/insert). Based on the ICP-MS results, the actual exposure dose (μg/cm^2^) of the Pd-NP in each cell model was calculated using the following formula:


$$\mathrm{Particle exposure dose}=\frac{\mathrm{Exact}\;\mathrm{dose}\;\mathrm{of}\;Pd-NP}{\mathrm{Insert}\;\mathrm{surface}\;\mathrm{area}:0.9cm^{2}}$$

#### Cell viability

##### Trypan blue staining

Trypan blue staining was used to estimate cell viability and to evaluate model quality. The cells were stained with 200 μL of 1:1 PBS diluted with 0.4% trypan blue solution for 1 min. After washing with PBS, viability was evaluated using bright-field microscopy (21, 28).

##### Lactate dehydrogenase (LDH) assay

The cell viability 24 h post-exposure was assessed with collected medium using an LDH assay kit from Thermo Fisher Scientific (Rockford, IL, USA), following the manufacturer’s instructions.

##### Apoptosis assay

The bronchial cells were trypsinized and collected by centrifugation at 1600 rpm for 10 min, followed by two washing steps with PBS. The cellular apoptosis rate was detected byBD Pharmingen™ PE Annexin V Apoptosis Detection Kit I (BD Biosciences, New Jersey, US). The cells were then resuspended in 100 μL of Annexin V binding buffer, and 5 μL of PE Annexin V and 7-amino-actinomycin (7-ADD) were added. After 15 min of incubation at room temperature in the dark, 400 μL of Annexin V binding buffer was added, and the cells were analyzed within 1 h using flow cytometry (21, 29).

#### ELISA

To assess the concentrations of inflammatory and tissue injury markers, CXCL-8 and MMP-9 were measured in the BM from both the sham and Pd-NP-exposed groups using the in-house ELISA method described previously (31). The commercially available antibody pairs MAB208 and MAF208 (R&D SYSTEMS®, UK) were used to measure CXCL-8, with a detection limit of 12.5 pg./mL. The concentration of MMP-9 was measured using a DouSet MMP-9 ELISA Kit (R&D Systems, UK). The detection limit of MMP-9 was 31.2 pg./mL (32). Absorbance read was using a Thermomax 250 reader and analyzed with Softmax® software 4.0 (Molecular Devices, Sunnyvale, CA, USA). An intra-assay CV <10% and an inter-assay CV <20% were accepted.

#### Quantitative real-time polymerase chain reaction (qRT–PCR)

The transcript expression of genes involved in oxidative stress, inflammation, and tissue injury/repair markers were assayed using qRT-PCR. The list of genes assessed, and the corresponding primer pairs used are provided in Supplementary Table S1. Total RNA were isolated from cells (Non-CB and CB-like models) following 24 h of exposure (sham and PD-NP at three different doses) using a RNeasy Mini Kit (Qiagen; *n* = 6) as described previously (28). The concentration of RNA was measured using a Nanodrop (ND1000 Technology). One microgram of mRNA was reverse transcribed to generate complementary DNA (cDNA) using a high-capacity RNA-to-cDNA kit (Life Technologies, Paisley, UK) and a thermal cycler (MycyclerTM, Bio-Rad). qRT–PCR was performed using an AB 7,500 System. The 20 μL of qRT–PCR mixture consisted of 10 μL of Fast SYBR^®^ Green Master Mix (Life Technologies, Paisley, UK), 200 nmol of each primer, 5 ng of cDNA, and nuclease-free water. Beta actin (ACTB) was used as the reference control. The expression of each target gene was quantified as the fold change following normalization to that of ACTB and sham-exposed Non-CB mucosa models. The results were calculated as 2-ΔCt (ΔCt = Ct (gene of interest) - Ct (beta actin).

### Statistics

Both Non-CB and CB-like mucosa models are well-differentiated tissue-like models, which contain multiple layers of cells including different cell types of unique distribution. Hence, in this study, every model (ie. both Non-CB and CB-like) are considered as a unique *in vivo*-like model with its own distribution of different cell types and the number of cells present might differ. Therefore, non-parametric statistical analysis was applied for the statistical evaluations. The results are expressed as median and interquartile ranges (25th-75th percentiles). Within Non-CB and CB-like models of 16HBE, comparisons between different Pd-NP exposure concentrations were assessed by the Friedman test followed by the Wilcoxon signed rank t test as a *post hoc* test. Comparisons between the Non-CB and CB-like models were performed by the Wilcoxon signed rank t test. A *p* value <0.05 was considered significant. All the data were analyzed using STATISTICA9 software (StatSoft, Inc., Uppsala, Sweden).

## Results

After 2 weeks of differentiation, airway mucosa models were characterized using microscopy images. Furthermore, the functional efficiency of 16HBE in the development of Non-CB and CB-like models were assessed by measuring the transcript expression of several inflammatory, oxidative stress, and tissue injury markers and were analyzed following exposure to aerosolized Pd-NP. Additionally, the secretion of MMP-9 and IL-8 from the basal medium was analyzed at the protein level after exposures.

### Model morphology

**Figure 2:**
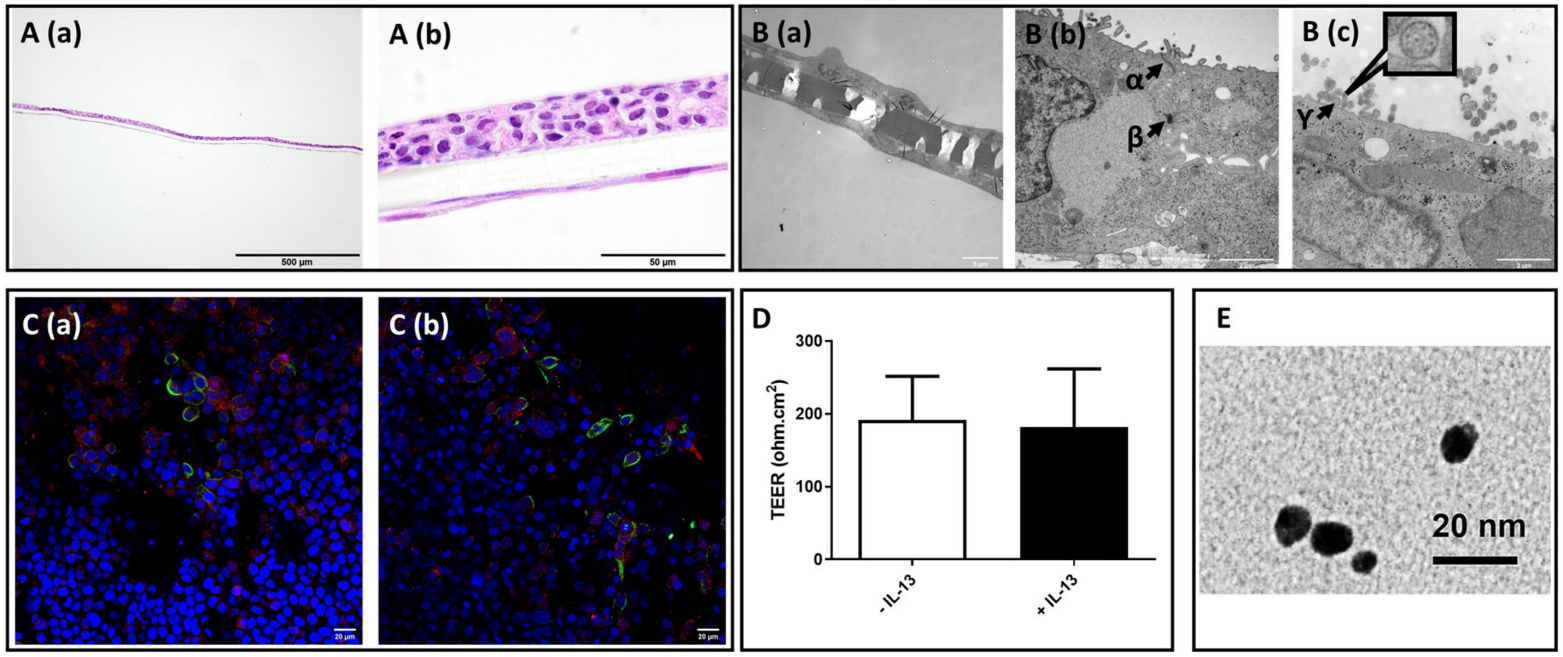
Morphological characterization of 16HBE mucosa models, transepithelial electrical resistance (TEER) value, and transmission electron microscopic (TEM) image of PN-NP. **(A)** Light microscope analysis of hematoxylin and eosin (H&E) staining of paraffin embedded cross-section of 2 weeks Non-CB models; Bar scale: 500 m A (a), 50 μm A (b). **(B)** Transmission electron microscope views of Non-CB model:B (a): Cross-sectional overview of the model with cells present on both sides of the membrane. Bar scale: 5 μm; B (b and c): High magnification images of the model cross-section, showing: *α*: tight junction; *β*:desmosome; *γ*: cross-section of the ciliary axoneme of 16HBEillustrating the 9 + 2 microtubule arrangement of motile cilia (high magnification figure), Bar scale: 2 μm. **(C)** Fluorescent immunostaining of α-tubulin (green), MUC5AC (red), and nuclear counterstaining (blue) of Non-CB (a) CB-like models (b), Bar scale: 20 μm. **(D)** Transepithelial electrical resistance (TEER) of Non-CB (blank) and CB-like (black) models; *N* = 8; Data presented as median and 25th-75th percentiles. **(E)** TEM image of PN-NP used in this experiment, Bar scale: 20 μm.

During cell propagation, 16HBE did not grow evenly throughout the culture plate surface but rather tended to grow in solitary or several colonies. It developed a furrow-like pattern after culture at the ALI site, and this pattern attenuated with time. Histological staining revealed constant bilayer formation for 16HBE and MRC-5 cells co-cultured on 0.4 μm semi-porous Transwell insert membranes (Figure 2A). 16HBE cells grew mostly in a stratified layer of 3–5 cuboidal cells, and MRC-5 cells formed a flat monolayer of cells spread out over the lower side of the membrane.

### Differentiation of 16HBE cells under ALI conditions and the formation of tight junctions

Characterization using TEM (Figure 2B) revealed the differentiation of 16HBE cells and the formation of ciliated cells after approximately 2 weeks of culture under ALI conditions. As shown in the TEM image, 16HBE cells formed visible intercellular adhesive structures, including supra-basal desmosomes and tight junctions, in the apical area. Confocal microscopy analysis in Figure 2C further confirmed the presence of both ciliated cells (*α*-tubulin: green) and mucus-producing cells (MUC5AC: red) in differentiated 16HBE cells cultured under ALI conditions, which is a typical morphological characteristic of the bronchial region of the lung. Additionally, 16HBE cells developed tight junctions in both the Non-CB and IL-13-treated CB-like models, with TEER values of 223 ± 105 *Ω*.cm^2^ and 198 ± 65 Ω.cm^2^, respectively (Figure 2D).

### Pd-NP exposure dose and characterization

In this study, both models were exposed to three doses of Pd-NP: 0.2 μg/cm^2^ (low), 0.3 μg/cm^2^ (medium), and 6 μg/cm^2^ (high). The actual exposure dose was calculated based on the formula in the methods section based on the ICP-MS results.

A high-magnification TEM image of the Pd-NP we used in this study, has been demonstrated in Figure 2E. Comprehensive details regarding the origin, purity, and physical characteristics of the Pd-NPs are available in our previous papers (21, 27).

### Effect of Pd-NP exposure on 16HBE cells in Non-CB and CB-like mucosa models

#### Cell viability

Cell viability was evaluated with trypan blue staining, colorimetric LDH assay and FACS after 24 h of exposure to Pd-NP. For FACS assays, we only performed with clean air (sham) and the high dose of Pd-NP exposed models. The first was also used to identify efficient models, which were subsequently excluded from the exposure studies. Both LDH and apoptosis assays confirmed that there was no difference in the cytotoxicity between the groups exposed to clean air (sham) and those exposed to Pd-NP (Supplementary Figure S1).

#### Pro-inflammatory effects

**Figure 3:**
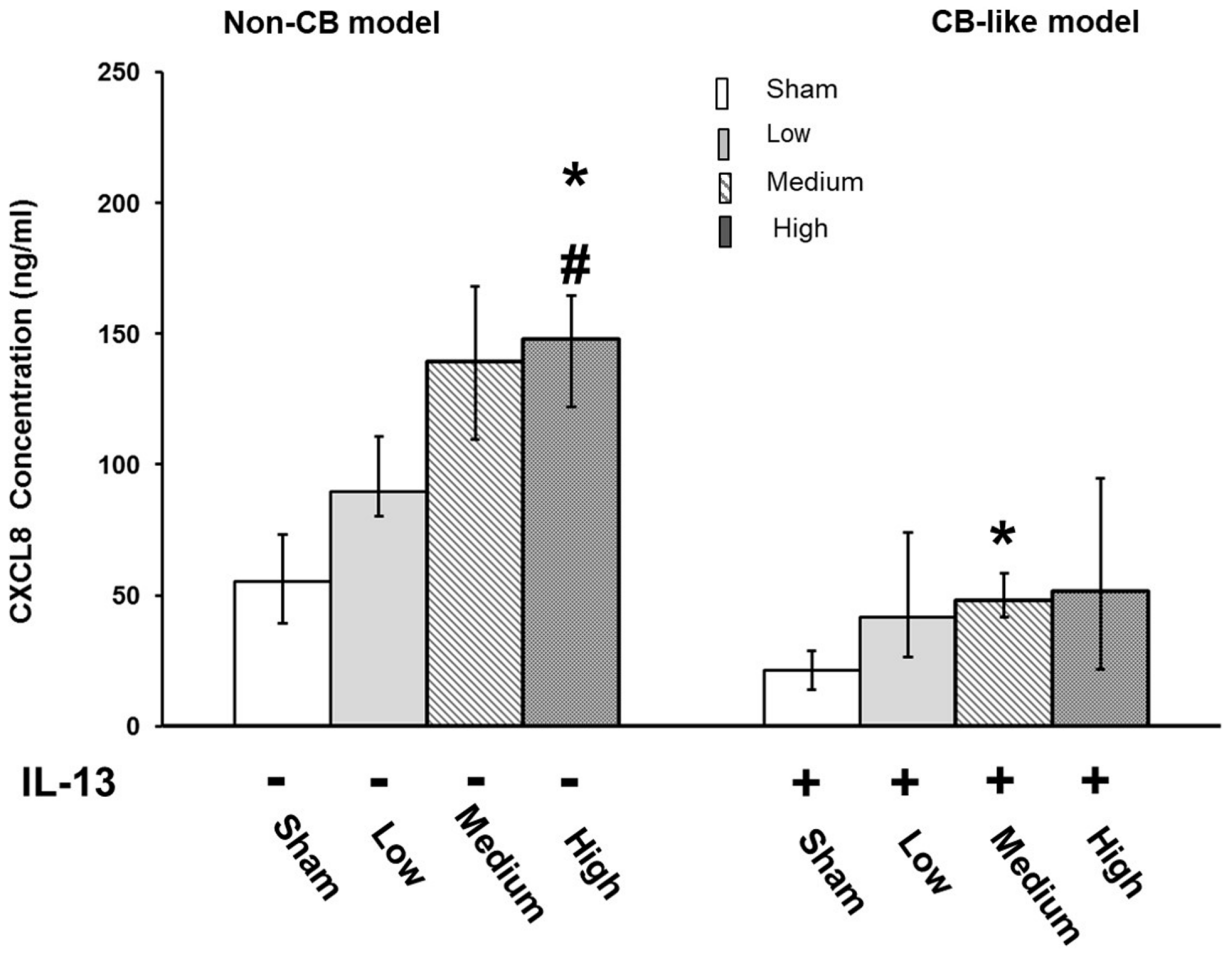
Palladium nanoparticle (Pd-NP) induced release of CXCL-8 in Non-CB and CB-like mucosa models after 24 h incubation following exposure to Pd-NP. The concentration of CXCL-8 was measured using ELISA from basal medium collected from both Non-CB and CB-like ALI models after incubation of 24 h following exposure to clean air (sham), low, medium, and high? Pd-NP doses. Data present as median and 25th-75th percentiles (*N* = 3, *n* = 9). *: *p* < 0.05 Sham; #: *p* < 0.05 low Pd-NP exposure.

In Non-CB mucosa models, CXCL-8 secretion increased in a dose-dependent manner at 24 h after exposure to Pd-NP; however, significantly increased secretion of CXCL8 was observed only at the highest dose of Pd-NP compared to that in the sham group (Figure 3, Left). Compared with that in the sham group, the secretion of CXCL8 in the CB-like mucosa model group remained significantly unchanged compared with that in the Non-CB mucosa model group (Figure 3, right).

**Figure 4:**
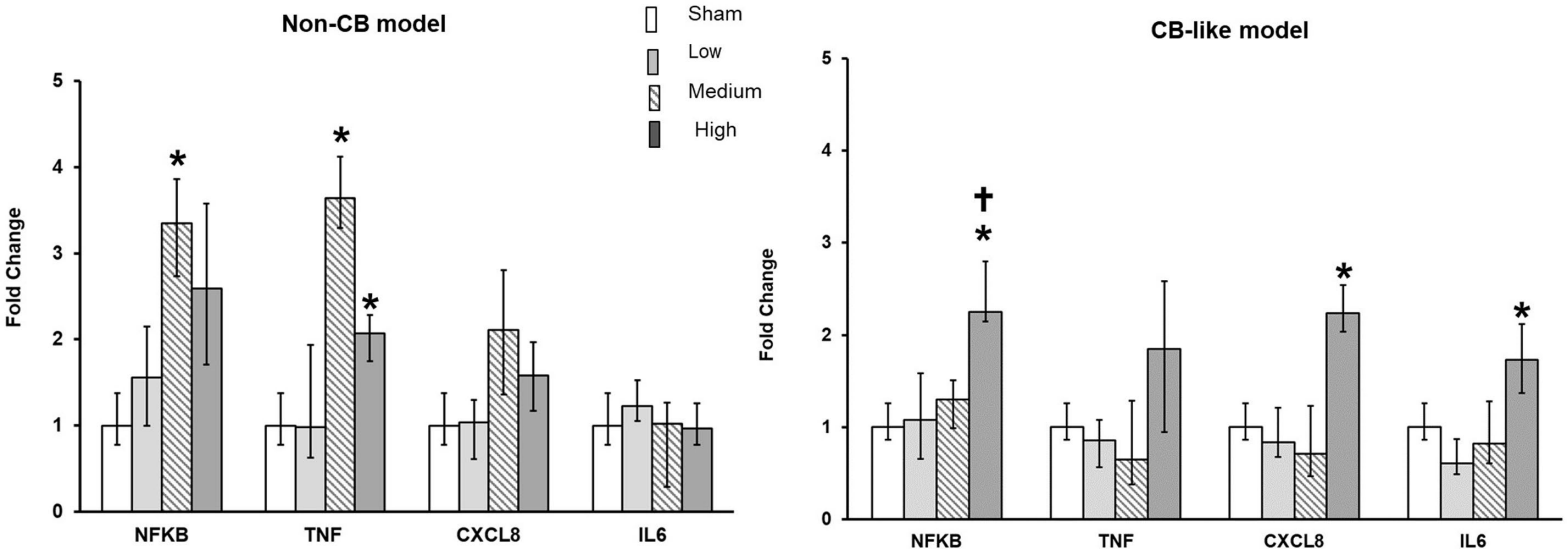
Palladium nanoparticles (Pd-NP) induced mRNA expression of inflammatory markers in Non-CB and CB-like mucosa models after 24 h incubation following exposure to PDNP. Fold change of *NFKB*, *TNF*, *CXCL8*, and *IL6* presented 24 h post exposure to clean air (sham), low, medium, and high Pd-NP doses. Data presented as median and 25th-75th percentiles (*N* = 3, *n* = 9). *: *p* < 0.05 vs. Sham.†: *p* < 0.05 vs. medium Pd-NP exposure.

The transcript expression levels of pro-inflammatory markers (*NFKB, CXCL8, TNFα,* and *IL6*) were assayed in both the Non-CB and CB-like models at 24 h postexposure to sham or aerosolized Pd-NP at all 3 tested doses. Compared with those in the sham group, the transcript levels of *NFKB* and *TNFα* were significantly higher in the Non-CB group at 24 h after Pd-NP exposure (at medium and high doses; Figure 4A). Furthermore, *CXCL8* and *IL6* expression remained unchanged in the Non-CB models, but in the CB-like models, *NFKB*, *CXCL8*, and *IL6* but not *TNFα* increased significantly 24 h after exposure to the high dose of Pd-NP (Figure 4B).

#### Oxidative stress response

**Figure 5:**
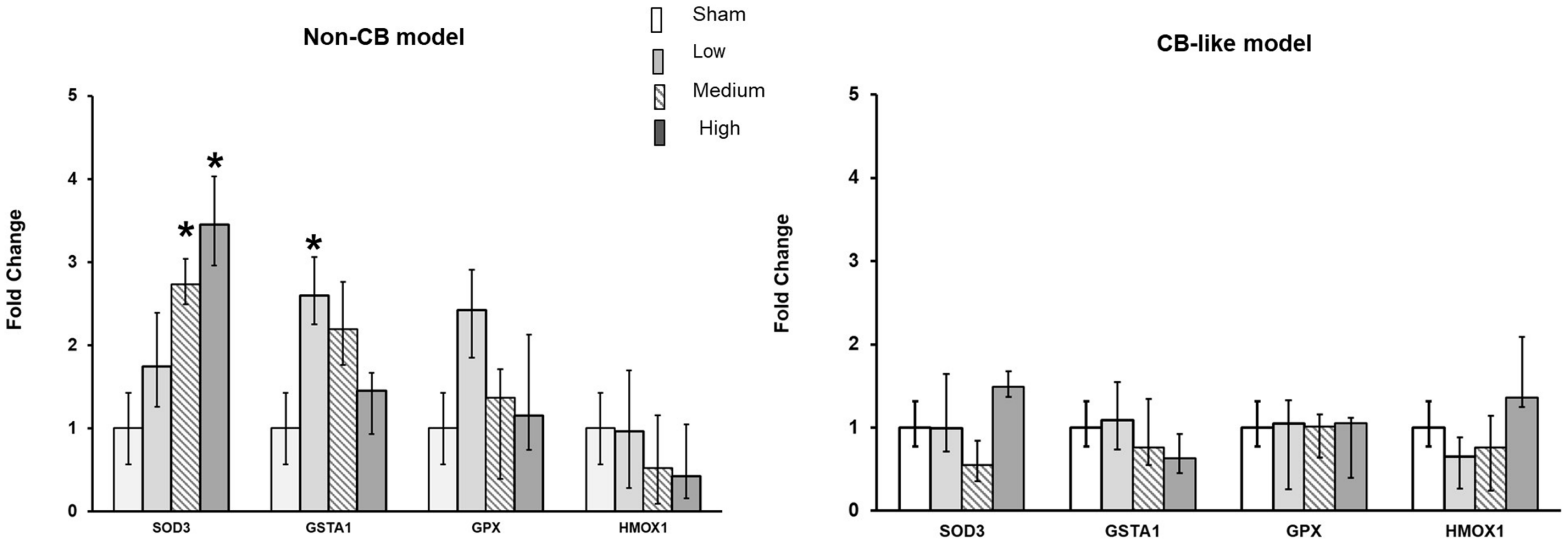
Palladium nanoparticles (Pd-NP) induced mRNA expression of oxidative stress markers in Non-CB and CB-like mucosa models after 24 h incubation following exposure to Pd-NP. Fold change of *SOD3, GSTA1, GPX, and HMOX1* presented 24 h post-exposure to clean air (sham), low, medium, and high Pd-NP doses. Data presented as median and 25th-75th percentiles (*N* = 3, *n* = 9). *: *p* < 0.05 vs. Sham.

Transcript expression analysis of oxidative stress markers (*SOD3, GSTA1, GPx*, and *HMOX1*) in both Non-CB and CB-like cells were performed at 24 h post-exposure to sham or 3 different doses of aerosolized Pd-NP. In the Non-CB cell models, *SOD3* expression clearly increased in a dose-dependent manner after exposure to Pd-NP, while *GSTA1* expression significantly increased (*p* < 0.05) only at the lowest dose of Pd-NP (Figure 5A). However, in the CB-like models, 24 h after exposure to Pd-NP, all oxidative stress markers remained unchanged except for *SOD3*, which increased following exposure to the highest dose of Pd-NP (Figure 5B).

#### Tissue injury/repair

**Figure 6:**
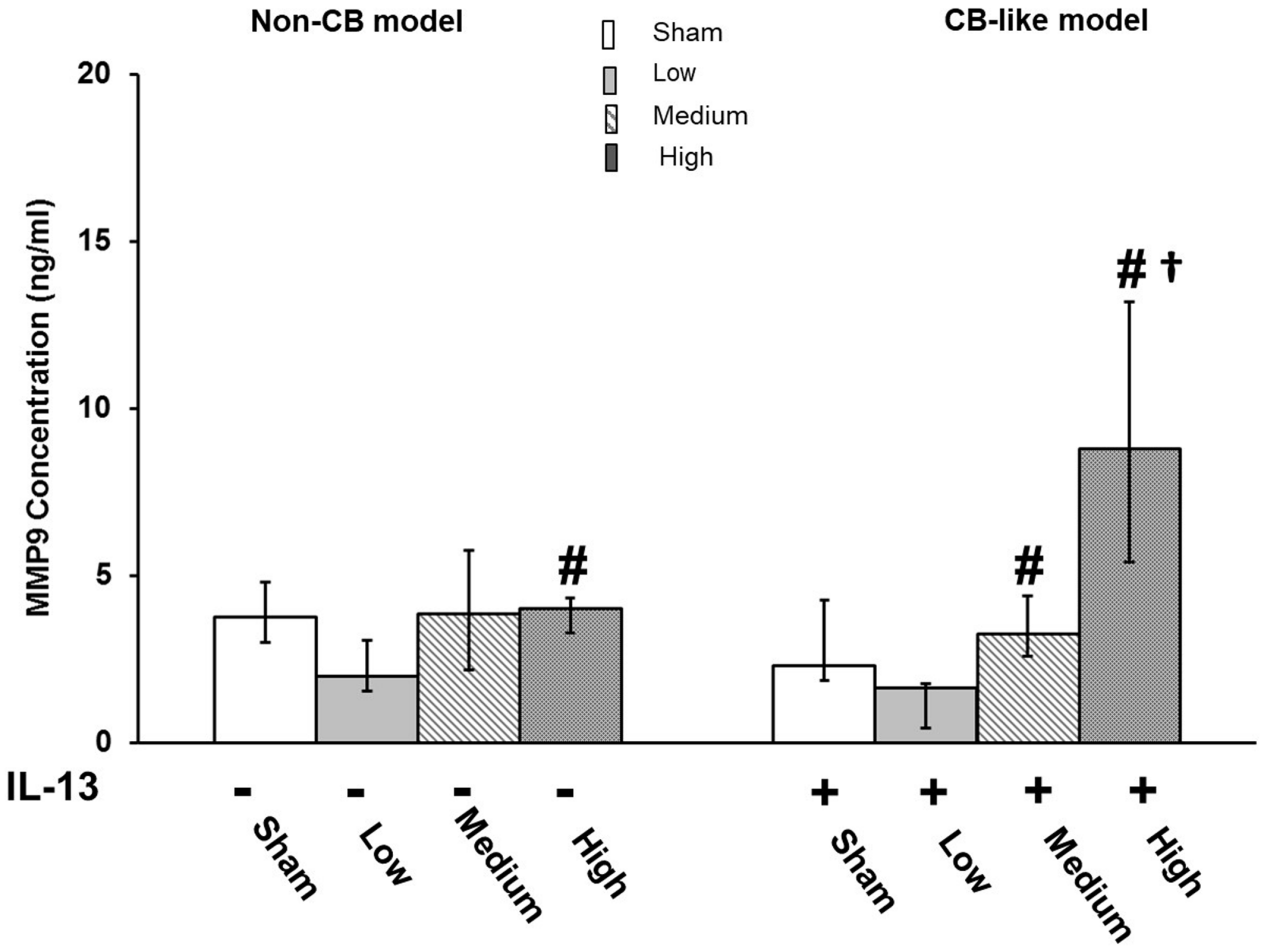
Palladium nanoparticles (Pd-NP) induced release of MMP9 in Non-CB and CB-like mucosa models after 24 h incubation following exposure to Pd-NP. The concentrations of MMP-9 were measured using ELISA from Basal Medium collected from both Non-CB and CB-like models after incubation of 24 h following exposure to clean air (sham), low, medium, and high Pd-NP doses. Data presented as median and 25th -75th percentiles (*N* = 3, *n* = 9). *: *p* < 0.05 vs. Sham.#: *p* < 0.05 vs. low Pd-NP exposure; †: *p* < 0.05 vs. medium Pd-NP exposure.

Pd-NP exposure did not affect the Non-CB models (Figure 6, left). However, in CB-like models, exposure to high concentrations of Pd-NP induced the release of MMP-9 compared to that in the sham, low-exposure, and medium-exposure groups (Figure 6, right).

## Discussion

In recent decades, different advanced physiologically relevant *in vitro* models have been established (21, 26, 28, 29, 33, 34). The checklist descriptors of the comparison between primary cells and cell lines under airlifted culture conditions were demonstrated in Supplementary Table S2. However, because of limited access and donor variations, high costs, and complicated handling processes, these primary cell-based models are less suitable for high-throughput applications from an economical, reproducible, and practical point of view. To address this limitation, we utilized the immortalized human bronchial epithelial cell line 16HBE (35) to establish both normal (Non-CB) and chronic bronchitis-like (CB) mucosa models. By combining with a sophisticated aerosol exposure platform, we exposed the models to varying concentrations of aerosolized Pd-NP. Functional assessment revealed increased secretion and expression of inflammatory biomarkers, as well as an induced oxidative stress response 24 h post-exposure to different doses of Pd-NP.

The lung’s primary defense mechanism, mucociliary clearance, relies on a diverse bronchial epithelium comprising ciliated, goblet, Clara, basal, and parabasal cells (16). Conventional submerged cultures lack this diversity, while air-liquid interface (ALI) cultures mimic *in vivo* conditions better, which promote the differentiation of bronchial epithelial cells into a mucociliary phenotype. In this study, we assessed 16HBE cell differentiation into ciliated and goblet cells via immunofluorescence staining of *α*-tubulin and MUC5AC, respectively. Contrary to previous studies (36, 37), which suggested negligible mucus production in mono-airlifted cultures, we observed both ciliated and mucus-producing cells. Our findings align with Nguyen Hoang et al. (25), who demonstrated mucus layer staining when 16HBE cells were co-cultured with dendritic cells on a collagen matrix with embedded fibroblasts and cultured at ALI. In our model, fibroblasts on basal side of the insert served as a feeding layer, emphasizing the need for co-culture conditions. Consistent with our previous study (26), which involved PBECs and THP-1-derived macrophages, thus this study underscores the critical role of cell–cell interactions in mimicking airway physiology, particularly the crosstalk between epithelial and mesenchymal cells.

Another role of the airway mucosa is the formation of a defense barrier to various inhaled xenobiotics (38). In this study, we demonstrated that 16HBE cells cultured under airlifted conditions produce a cell barrier with a morphology similar to that of the airway epithelium (as shown by H&E staining, immunofluorescence, and TEM images). The TEER measurements showed values around 250 *Ω* cm^2^ for both Non-CB and IL-13-treated CB-like models, which are consistent with previous studies using ALI cultures (21). Submerged cultures typically exhibit 70% greater resistance (39, 40), but Kidney et al. (41) reported a mean resistance of 100 Ω in surgical specimens of human bronchi, therefore, the lower TEER values in our 16HBE ALI model better mimic lung mucosa models with primary cells and *in vivo* conditions. Although TEM images revealed the presence of junctional complexes in our 16HBE ALI models, future investigations may benefit from performing immunofluorescence staining of tight junction proteins, such as Zonula occludens-1and occludin/*β*-catenin to provide additional information about barrier integrity (42).

Animal studies have indicated that for both acute exposure and sub-chronic exposure, IL-6 levels are increased in the serum of rats after intravenous administration of Pd-NP (43, 44). In our previous publications, we showed immune effects on lung epithelial cells after Pd-NP exposure (21, 27). In submerged culture conditions of PBECs and A549 cells, we showed that solution-engineered Pd-NP and Pd–Al_2_O_3_ NP exert concentration-dependent cytotoxicity (27). Additionally, we have shown a profound inflammatory effect, for instance, by inducing the expression of different biomarkers (like IL-8, MMP-9) following the exposure of PBECs to aerosolized Pd-NP under ALI conditions (21). Since the cellular response is associated with exposure methods, it is difficult to perform a direct scientific comparison between submerged and ALI-exposed models. For instance, adding NPs directly to a submerged cell culture medium can modify the properties (agglomeration, partial dissolution, etc.) of NPs, which will alter the interaction between NPs and cells and further lead to mismeasurement of the outcomes (45). However, both previous and present studies showed a similar immune response tendency, even at different exposure dose levels. Exposure to upto10 μg/ml Pd-NP had pronounced effects on the secretion of inflammatory biomarkers such as IL-8 in PBECs and A549 cells under submerged conditions. Interestingly, a similar response was observed in PBECs following exposure to aerosolized 200–650 ng/cm^2^ Pd-NP under ALI conditions. Similarly, we found that in 16HBE Non-CB models, there was a dose-dependent increase in IL-8 secretion at 24 h after exposure to Pd-NP aerosols, especially with significant induction in the high-concentration exposure group. The mRNA expression of inflammatory genes such as *NF-κB* and *TNFα* were also significantly increased in Non-CB models cultured at the ALI site 24 h after high-dose exposure to Pd-NP.

The mechanism underlying Pd-NP induced oxidative stress involves the generation of ROS through redox reactions catalyzed by the nanoparticles (46). The oxidative stress can trigger a cascade of cellular responses, including activation of stress-responsive signaling pathways, DNA damage repair mechanisms, and eventually causes cell apoptosis (46). In this study, for Non-CB lung mucosa models with 16HBE cells, we detected the expression of antioxidative components such as *SOD3*, which clearly responded to exposure to Pd-NP in a dose-dependent manner. Numerous studies have reported similar observations across various cell types, including human lung epithelial cells and other cells such as Caco-2 (47), A549 (48), H1229 (6), THP-1 cells (49), human skin cells (50) and human eosinophils (51). The ability of Pd-NP to induce oxidative stress and subsequent cytotoxicity have implications for various physiological processes and pathological conditions. These may raise concerns regarding their long-term implications for human health, particularly in occupational settings where exposure to these nanoparticles may be more prevalent (5). Several adverse health effects are caused by occupational exposure to Pd. For instance, airborne palladium (Pd) concentrations during various tasks in a precious metal refinery were found to range from 0.3 to 32 μg/m^3^, with a mean concentration of 4.99 μg/m^3^ (*n* = 10) (52). In catalyst production plants, the highest level of Pd in airborne matter can reach 7.9 μg/m^3^ (53). Although occupational exposure limits (OELs) for Pd nanoparticles are not well-defined, Finland has established OELs (8 h) for Pd compounds at 0.5 mg/m^3^ for insoluble Pd and 1.5 μg/m^3^ for soluble Pd (54).

IL-13 is a pleiotropic cytokine that causes goblet cell hyperplasia and excessive mucus production *in vitro*, as well as airway hyperresponsiveness, allergic inflammation, and tissue remodeling *in vivo*. These pathological features of human respiratory disorders include chronic bronchitis, asthma, and COPD (55–57). In our previous studies (21, 33), we successfully developed chronic bronchitis-like mucosa models including an increased number of mucus-producing cells by treating the PBEC-ALI models with 1 ng/mL IL-13. It is worth mentioning that we use the term “chronic bronchitis-like” to reflect specific aspects of the phenotype we are modeling. However, other diseases such as asthma, emphysema, and pulmonary fibrosis may also be implicated. We have previously demonstrated (21, 33) that CB-like models are more sensitive to Pd/carbon nanoparticle exposure than Non-CB mucosa models, including PBECs cultured at the ALI site. Here in this study, in the CB-like models, the mRNA expression of *NFκB, CXCL8*, and *IL6* increased significantly 24 h after exposure to the high dose of Pd-NP. However, the levels of oxidative stress markers remained unchanged. MMP-9 is involved in lung epithelial wound repair by remodeling the provisional ECM and controlling the migration of repairing cells (58). We did not find a significant difference in the secretion of MMP-9 between Non-CB and CB-like models, but high-dose exposure to Pd-NP increased MMP-9 secretion in CB-like models. Although noble metal NPs such as Au and Pt have been proven to inhibit the activity of MMP9 in RAW264.7 cells, IL-13 treatment increased the production of MMP-9 in different types of cells (59, 60). In addition, in our previous study (33), we showed that *MMP9* mRNA expression was increased by exposure to carbon nanoparticles and was significantly greater in CB-like PBEC-ALI models than in Non-CB models. Taken together, the results from CB-like ALI models established with 16HBE cells showed a pattern similar to that of primary cell models. Therefore, 16HBE-based cell models, including epithelial cell lines, which are easier to access, can be useful as alternative Non-CB and disease-like models for nanoparticle risk assessment.

Cell models have inherent limitations, including the absence of all cell types present *in vivo*. For instance, immune cells are not present in this model. Also, our study the lack of investigation into the role of fibroblasts in modulating the 16HBE phenotype, particularly in comparison to cultures without fibroblasts, and their response to IL-13 stimulation. Fibroblasts are essential components of the epithelial microenvironment, significantly influencing epithelial cell behavior and response to cytokines such as IL-13 (61, 62). Understanding the interactions between fibroblasts and epithelial cells in our co-culture models are crucial, as fibroblasts may alter the epithelial response to IL-13, potentially affecting mucus production and other phenotypic changes. Therefore, future research should focus on incorporating a greater variety of cell types into the models and elucidating the interactions between different types of cell within the co-culture system. Additionally, although transwell models are valuable for studying certain aspects of pulmonary toxicity caused by xenobiotics, they do not fully replicate the dynamic and complex interactions observed *in vivo*. The static nature and simplified structure of transwell models limit their ability to mimic the full spectrum of cellular interactions and responses present*in vivo*. Besides, in this study, although we analyzed markers such as HMOX1, SOD3, GPx, and GSTA1 to understand the oxidative stress response induced by Pd-NP exposure, we did not dig into detailed mechanistic pathways, such as the regulation of NF- kB activation or the modulation of the GSH/SH ratio. Our primary focus was on demonstrating that our models can provide insights into cellular responses relevant to pulmonary health, rather than conducting detailed mechanistic pathway analyses. Nevertheless, our study lays a foundation for future investigations aimed at integrating detailed mechanistic studies to comprehensively evaluate nanoparticle toxicity.

## Conclusion

Both normal (Non-CB) and chronic bronchitis-like (CB-like) models effectively replicate crucial key features of an *in vivo*-like physiochemical defense barrier. Our models also suggest the necessity of co-culture of 16HBE with fibroblasts to successfully mimic *in vivo* airway mucosa. In addition, we evaluated the potential functionality of the models by studying the mechanisms of particle-induced inflammation, the oxidative stress response, and lung injury/repair. 16HBE is a well-known cell line which form extensive tight junction while having less mucus producing ability compared to primary bronchial epithelial cells (PBECs) (63), but theyare easy to handling and growing and can replace primary cells for large-scale and/or high-throughput toxicity screening and profiling. The 16HEC mucosa models we established can contribute to a better risk assessment of inhaled particles and improve the understanding of pulmonary diseases. Furthermore, they can also help to reduce/refine animal experiments and thereby save costs and bridge the translational gap between currently available models and real-life human situations.

## Data Availability

The original contributions presented in the study are included in the article/Supplementary material, further inquiries can be directed to the corresponding authors.
