# Supplementary material for: Cell line-based in vitro models of normal and chronic bronchitis-like airway mucosa to study the toxic potential of aerosolized palladium nanoparticles
Source: Front Med (Lausanne). 2024 Oct 8;11:1422792. doi: 10.3389/fmed.2024.1422792 (PMC11493715; doi:10.3389/fmed.2024.1422792)
Supplement: Supplementary file 1 [file Data_Sheet_1.pdf]

Table S1. Primers used for quantitative real-time PCR (qPCR)

Gene name	Gene Symbol	Forward Primer 5'-3'	Reverse Primer 3'-5'
Beta (β)-Actin	ACTB	CTGGGACGACATGCAGAAAA	AAGGAAGGCTGGAAGAGTGC
Nuclear factor kappa-light-chain-enhancer of activated B cells	NFKB	AAGAGGAGGTTTCGCCACCG	TTGCAGATTTTGACCTGAGGGT
Tumor necrosis factor alpha	TNFα	AGCCCATGTTGTAGCAAACC	ACATTGGGTCCCCCAGGATA
C-X-C Motif Chemokine Ligand 8	CXCL8	GCTCTGTGTGAAGGTGCAGTT	GGCACAGTGGAACAAGGACT
Interleukin 6	IL6	ACCCCCAGGAGAAGATTCCA	CACCAGGCAAGTCTCCTCATT
Superoxide Dismutase 3	SOD3	ACGCTGGCGAGGACGACCTG	GCTTCTTGCGCTCTGAGTGCTC
Glutathione S Transferase alpha 1	GSTA1	TGCAGCTGGAGTAGAGTTTG	ATGGGCACTTGCTGGAACAT
Glutathione Peroxidase1	GPX1	GTGCTCGGCTTCCCGTGCAAC	CTCGAAGAGCATGAAGTTGGGC
Heme oxygenase 1	HMOX1	TTCAAGCAGCTCTACCGCTC	GGGGGCAGAATCTTGCACTTT

Table S2. The checklist descriptors of the comparison between primary cells and 16HBE under airlifted culture conditions

	3D cell culture parameter	Primary cells [7-9]	Cell line (16HBE)[10, 11]
Cells	Cell types	Ciliated cells, goblet cells, Clara cells, basal cells	Ciliated cells, goblet cells, basal cells
	Cell availability	Limited	Unlimited
	Cell identity	Variance between different donors; donor's health affects model characteristics	Immortalized; standardized characterization
	Ethics	Ethical permission may be required	Not required
Cell culture manipulation	Handling	Require more experience; cells sensitive to mistreatment	Easier to work with
	Culture medium	Serum-free medium; specific growth factors; may need optimization	Standardized medium
	Growth time	4 weeks before exposure	4 weeks (with extension possibility) before exposure
	Cost	High	Low
Biological functions/features of culture	Quality standards	Differ between labs; usually depends on experiment's purpose	Standardized quality control
	Culture morphology	Pseudostratified structure; 3-5 layers of cells	Cobblestone morphology; 3-5 cell layers

	Culture functionality	Polarized mucociliary differentiated airway epithelial cell layer	Epithelial layer expresses mucins and some cilia; barrier function
	Relevance to human tissue	High; maintains morphology, gene expression, key metabolic enzymes	Low
Assay validation for toxicity/efficacy testing	Reproducibility of the results	Low (due to donor-to-donor variations)	High
	Throughput	Low	High

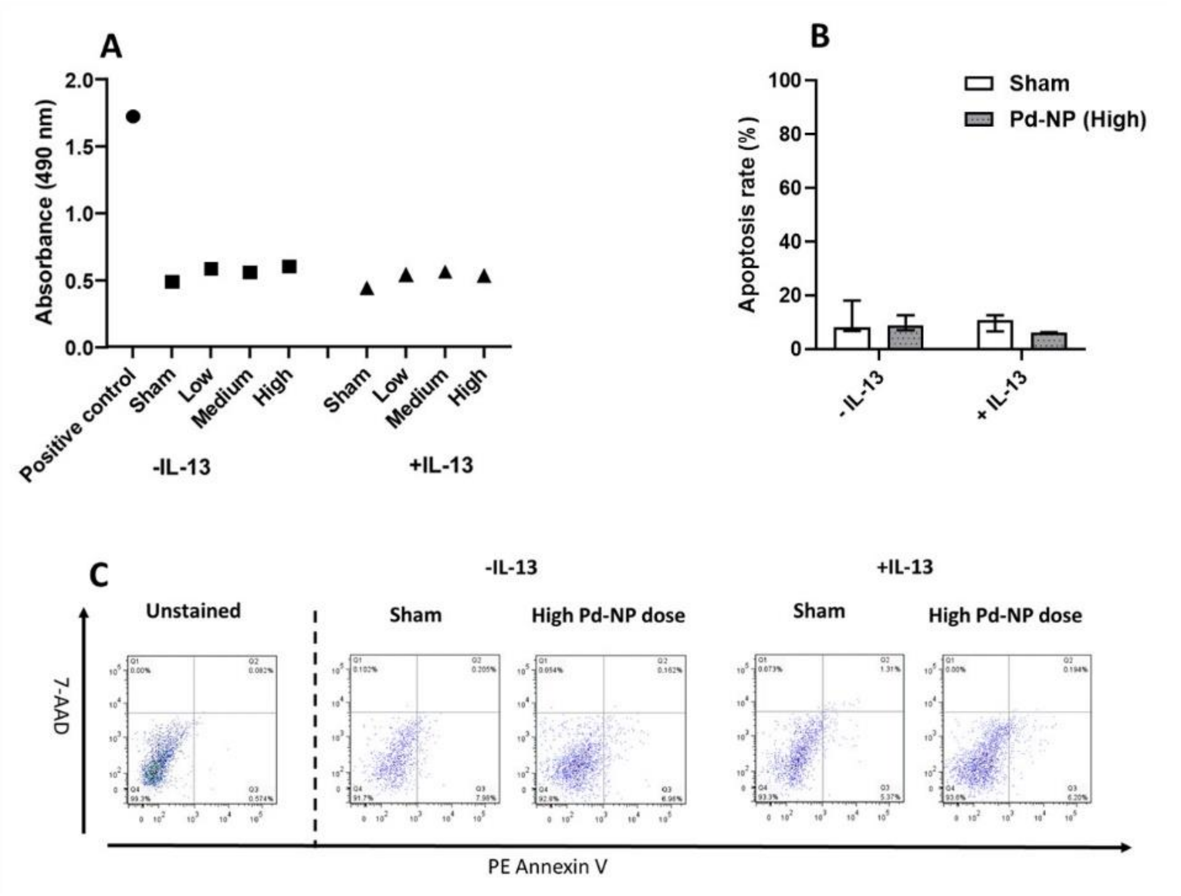

Figure S1. Palladium nanoparticle (Pd-NP) induced cytotoxicity and apoptosis rate in Non-CB and CB mucosa models after 24h incubation following exposure to Pd-NP.

The cytotoxic effect was measured by colorimetric Lactate Dehydrogenase assay after 24 h of exposure to the clean air (sham) and all Pd-NP doses, positive Control (N=2) (A). The apoptosis rate was detected by FACS after 24 h of exposure to the clean air (sham) and high Pd-NP dose. Data presented as median and 25th -75th percentiles (N=3) (B). The gating strategy used to identify apoptotic cells (C).

References

1. Fothergill, S.J.R., D.F. Withers, and F.S. Clements, *Determination of Traces of Platinum and Palladium in the Atmosphere of a Platinum Refinery: By a Combined Chemical and Spectrographic Method.* British Journal of Industrial Medicine, 1945. **2**(2): p. 99-101.
2. Violante, N., et al., *Assessment of workers' exposure to palladium in a catalyst production plant.* J Environ Monit, 2005. **7**(5): p. 463-8.
3. *HTP-arvot 2014 Haitallisiksi tunnetut pitoisuudethttps*. 2014; Available from: https://julkaisut.valtioneuvosto.fi/bitstream/handle/10024/162457/STM_2020_24_J.pdf.
4. McCarrick, S., H.L. Karlsson, and U. Carlander, *Modelled lung deposition and retention of welding fume particles in occupational scenarios: a comparison to doses used in vitro.* Arch Toxicol, 2022. **96**(4): p. 969-985.
5. Pleil, J.D., M. Ariel Geer Wallace, M.D. Davis, and C.M. Matty, *The physics of human breathing: flow, timing, volume, and pressure parameters for normal, on-demand, and ventilator respiration.* J Breath Res, 2021. **15**(4).
6. Londahl, J., et al., *Measurement techniques for respiratory tract deposition of airborne nanoparticles: a critical review.* J Aerosol Med Pulm Drug Deliv, 2014. **27**(4): p. 229-54.
7. Ji, J., et al., *Development of Combining of Human Bronchial Mucosa Models with XposeALI(R) for Exposure of Air Pollution Nanoparticles.* PLoS One, 2017. **12**(1): p. e0170428.
8. Baxter, A., et al., *Targeted omics analyses, and metabolic enzyme activity assays demonstrate maintenance of key mucociliary characteristics in long term cultures of reconstituted human airway epithelia.* Toxicol In Vitro, 2015. **29**(5): p. 864-75.
9. Dvorak, A., et al., *Do airway epithelium air-liquid cultures represent the in vivo airway epithelium transcriptome?* Am J Respir Cell Mol Biol, 2011. **44**(4): p. 465-73.
10. Cozens, A.L., et al., *CFTR expression and chloride secretion in polarized immortal human bronchial epithelial cells.* Am J Respir Cell Mol Biol, 1994. **10**(1): p. 38-47.
11. Forbes, B., A. Shah, G.P. Martin, and A.B. Lansley, *The human bronchial epithelial cell line 16HBE14o- as a model system of the airways for studying drug transport.* Int J Pharm, 2003. **257**(1-2): p. 161-7.
